# Immunosenescence in atherosclerosis: A role for chronic viral infections

**DOI:** 10.3389/fimmu.2022.945016

**Published:** 2022-08-17

**Authors:** Atefe Ghamar Talepoor, Mehrnoosh Doroudchi

**Affiliations:** Department of Immunology, School of Medicine, Shiraz University of Medical Sciences, Shiraz, Iran

**Keywords:** immunosenescence, cell senescence, atherosclerosis, inflammaging, viral infections

## Abstract

Immune system is a versatile and dynamic body organ which offers survival and endurance of human beings in their hostile living environment. However, similar to other cells, immune cells are hijacked by senescence. The ageing immune cells lose their beneficial functions but continue to produce inflammatory mediators which draw other immune and non-immune cells to the senescence loop. Immunosenescence has been shown to be associated with different pathological conditions and diseases, among which atherosclerosis has recently come to light. There are common drivers of both immunosenescence and atherosclerosis; e.g. inflammation, reactive oxygen species (ROS), chronic viral infections, genomic damage, oxidized-LDL, hypertension, cigarette smoke, hyperglycaemia, and mitochondrial failure. Chronic viral infections induce inflammaging, sustained cytokine signaling, ROS generation and DNA damage which are associated with atherogenesis. Accumulating evidence shows that several DNA and RNA viruses are stimulators of immunosenescence and atherosclerosis in an interrelated network. DNA viruses such as CMV, EBV and HBV upregulate p16, p21 and p53 senescence-associated molecules; induce inflammaging, metabolic reprogramming of infected cells, replicative senescence and telomere shortening. RNA viruses such as HCV and HIV induce ROS generation, DNA damage, induction of senescence-associated secretory phenotype (SASP), metabolic reprogramming of infected cells, G1 cell cycle arrest, telomere shortening, as well as epigenetic modifications of DNA and histones. The newly emerged SARS-CoV-2 virus is also a potent inducer of cytokine storm and SASP. The spike protein of SARS-CoV-2 promotes senescence phenotype in endothelial cells by augmenting p16, p21, senescence-associated β-galactosidase (SA-β-Gal) and adhesion molecules expression. The impact of SARS-CoV-2 mega-inflammation on atherogenesis, however, remains to be investigated. In this review we focus on the common processes in immunosenescence and atherogenesis caused by chronic viral infections and discuss the current knowledge on this topic.

## Immunosenescence is a widespread phenomenon

Ageing is defined as progressive physiological changes along with decline of biological functions in a cell, organ, or the total organism which is associated with increased risk of debility, disease, and death (1). Inflammaging is a key underlying mechanism in processes leading to aging and ageing-related diseases. The term “inflammaging” refers to a chronic, low level systemic inflammation that results in overstimulation of the immune system and elevated mortality and morbidity in elderly individuals (2). Growing evidence suggests the existence of interplay between ageing and immune system alterations, which is called immunosenescence (3). Therefore, immunosenescence is referred to the gradual deterioration of immune parameters as a consequence of ageing process. The important hallmarks of immunosenescence are the followings: [1] reduced frequencies of naïve T cells, [2] alternation in CD4:CD8 ratio, [3] defective response to new antigens, [4] accumulation of short-lived memory T cells, [5] impaired calcium-mediated signaling, and [6] inflammaging (4–6). Immunosenescence affects both the ability to respond to infectious agents as well as development of appropriate and long-term immune responses (7). It has also been shown that immunosenescence affects adaptive immune system more than the innate immune system (8). The main characteristics of immunosenescence with respect to the innate immunity include reduced number of circulating CD14^+^ CD16^−^classical monocytes, macrophages and dendritic cells (DCs), reduced chemotaxis and phagocytosis of macrophages and neutrophils, defective reactive oxygen species (ROS) production by neutrophils, increased neutrophil susceptibility to apoptosis, elevated levels of inflammatory cytokines and chemokines, lower expression level of major histocompatibility complex (MHC) class II by DCs and macrophages, increased number of natural killer (NK) cells, decreased NK cells cytotoxicity and impaired antigen presentation by DCs (9–17). In terms of adaptive immunity, immunosenescence can result in reduced naive CD4^+^ and CD8^+^T cells pools, restricted T cell receptor (TCR) repertoire, loss of CD28 expression by T cells, increased frequencies of effector (T_Eff_) and effector memory T (T_EM_) cells, decreased accumulation of central memory T (T_CM_) cells, elevated accumulation of memory B cells, restricted B cell receptor (BCR) repertoire, impaired class-switching and somatic recombination of B cells and defective antibody responses to antigens (17–19). It has been demonstrated that immunosenescence, through stimulating proinflammatory factors and inflammaging, can drive cellular senescence in other tissues and development of age-related diseases, such as infections, cancer, autoimmune disorders and chronic inflammatory and metabolic diseases (20, 21) in the elderly. Although immunosenescence and cellular senescence are slightly different in mechanism, they share several similarities. In the following sections we will describe different properties, drivers and regulators of cellular senescence and immunosenescence with the focus on atherosclerosis.

## Overview of cellular senescence

Different types of somatic cells isolated from mammalian tissues undergo multiple proliferations before they stop growing. For the first time in 1961, Hayflick and Moorhead discovered that human fibroblast cells proliferation potency is limited upon serial culture, and they called this phenomenon “cellular senescence” (22).

Cellular senescence is an irreversible cell cycle arrest in the G1 phase, in which the senescent cells remain metabolically active but they hold a growth arrested status (23). Indeed, upregulation of the p16, p21 and p53 cell cycle inhibitors lead to the irreversible cell cycle arrest in the senescent cells (2, 24). Hence, cellular senescence is different from other non-dividing processes such as quiescence, exhaustion or terminally differentiation by several markers and morphological changes (24–26). The replicative senescence (RS), oncogene-induced senescence (OIS), genotoxicity-induced senescence (GIS), developmental senescence and tissue repair senescence are considered different types of cellular senescence in human (25, 27, 28).

In general, senescence in any cell type is accompanied by phenotypic alterations including but not limited to cytoplasm enlargement, flattened, vacuolated, and multinucleated cell morphologies, depending on cell type, genetic background, and the type of senescence inducing stimuli (29). In addition, senescent immune cells show reduced DNA replication, chromatin remodeling, metabolic reprogramming, increased resistance to apoptosis, and increased expression of senescence-associated β-galactosidase (SA-β-Gal) as well as producing a set of inflammatory secretome (30–32). Moreover, immunoscenesent cells downregulate surface markers like CD27, CD28, CCR7 and CD45RO, while they upregulate the Killer cell lectin-like receptor subfamily G (KLRG-1), CD57, PD-1 and CD153 (29, 33–35).

Senescent immune and non-immune cells produce a combination of inflammatory factors, called senescence-associated secretory phenotype (SASP) (36). SASP factors consist of several interleukins (ILs) including IL-6 and IL-1, and chemokines such as CXCL-4,-5, -6, -12 and CCL-2, -3, -7, -8, -13, -16, -20 and -26, proteases, growth factors, bioactive lipids and extracellular vesicles which are involved in immune cell migration, innate and adaptive immune responses, modification of the extracellular matrix (ECM), cell cycle arrest and phagocytosis (24, 37, 38). Particularly, secreted SASP factors act as paracrine and autocrine mediators that promote inflammatory responses as well as defective tissue remodeling which ultimately may lead to chronic autoimmune disorders and different cancers (39–41). A variety of transcription factors and signaling pathways like nuclear factor kappa B (NF-κB) and beta CCAAT/enhancer– binding protein (42, 43), DNA damage (44), p38α mitogen-activated protein kinase (MAPK) (45), mammalian target of rapamycin (mTOR) (46, 47), and mixed lineage leukemia 1 (48) and GATA4 (49) regulate the key effectors of the SASP.

Two major tumor suppressor pathways, p16^INK4A^/pRB and p53/p21^WAF1/CIP1^, through upstream regulators and downstream effectors along with extensive inter-pathway crosstalk, are responsible for the stable growth arrest in the senescence (50, 51). The p16^INK4A^/pRB pathway mainly acts as a negative regulator of cell cycle progression by downregulating cyclin-dependent kinase (CDK) 4/6 activity. As a result, hypo-phosphorylated Rb binds to E2F complex and leads to repression of E2F target gene transcription required for cell cycle progression (52). P53 is activated in response to DNA damage and induces transcription of cyclin-dependent kinase inhibitors (CDKIs) and p21^CIP1^ which triggers growth arrest and cellular senescence (31, 53). Therefore, overexpression of these four components including p53, p21^CIP1^, p16^INK4A^ and PRB are sufficient to induce and maintain cellular senescence.

## Senescence in physiological and pathological processes

Several studies have shown that cellular senescence playsphysiological roles in the embryonic and later life development, wound healing, tissue repair and protective response to stress (54–57). In addition, as senescent immune and non-immune cells accumulate in multiple tissues, they may contribute to regulate the non-pathological and pathological senescence-related states (39). In some events, acute senescence exhibits protective effects and thus prevents the progression of the diseases. For instance, during early stages of tumor, the activation of oncogenes stimulates cellular senescence in an attempt to inhibit cell growth and tumor progression (58). In other circumstances, chronic and aberrant accumulation of senescent cells in tissues generates a pro-inflammatory environment that affects the onset, development or progression of several senescence-associated diseases, such as atherosclerosis, cardiovascular diseases (CVDs), cancer, hepatic steatosis, Alzheimer’s disease, fibrotic pulmonary disease, osteoarthritis, glaucoma, type 2 diabetes and renal dysfunction (59–64).

## Senescence and immunosenescence in atherosclerosis

Atherosclerosis is a chronic inflammatory and age-associated condition of medium to large arteries which is linked to the progression of CVDs, such as abdominal aortic aneurysm (AAA), coronary artery disease (CAD), peripheral artery disease (PAD), heart failure (HF) and ischemic strokes (65). Endothelial abnormalities, vascular inflammation, adhesion molecules expression, cytokines and chemokines secretion, immune cells infiltration, monocytes entry into intima, engulfment of oxidized low density lipoprotein (Ox-LDL) and foam cell formation result in atherosclerotic plaque formation. Subsequently, aberrant efferocytosis, foam cells apoptosis, migration of vascular smooth muscle cells (VSMCs) from media to intima, degradation of ECM and extensive coagulation lead to the rupture and erosion of atherosclerotic plaques, vessel occlusion and the risk of death (66–68).

Several findings showed that cellular senescence in the immune and non-immune cells are involved in the early and advanced stages of atherosclerosis (69). Certainly, accumulation of the senescent endothelial cells (ECs) (70, 71), VSMCs (72, 73), monocytes (74), macrophages and foam cells (71, 75), fibroblasts (74) and T cells (76, 77) influence atherosclerosis process by necrotic core enlargement, ECM destruction, atherosclerotic plaque calcification, intra- plaque angiogenesis and its rupture (78). ECs, as the lining of the intima layer, play major roles in the maintenance of the integrity of arteries. Thus, EC dysfunction and senescence, which can cause elevated blood pressure, increased coagulation and angiogenesis as well as elevated inflammatory response, contribute in progression of atherosclerosis (79, 80). It is not clear, however, if they take part in onset of atherosclerosis, as well. Senescent ECs are frequently found in atherosclerotic plaques of elderly patients and senescent cells have been proposed to be a major contributor to development of vascular diseases (81, 82). ECs in advanced human plaques show evidence of senescence, including telomere shortening, increased p53/p21 signaling pathways and elevated SA-β-GAL activity (83, 84). Senescent ECs are associated with increased levels of ROS, inflammatory cytokines and extracellular vesicles secretion as well as vascular calcification (85, 86). Furthermore, it has been shown that senescent ECs produce lower levels of nitric oxide (NO) and prostacyclin and thereby affect vascular homeostasis (87). On the other hand, secretion of inflammatory mediators and ROS by senescent ECs, retention of Ox-LDL in the arterial intima, adhesion of monocytes to endothelium, activation of NF-κB signaling and production of extracellular vesicles are potent promoters of SASP-mediated senescence and possibly play a role in the atherosclerotic plaque development (88–92). Current studies, however, do not provide information on the senescence of ECs or immune cells in early onset atherosclerosis and cardiovascular diseases.

VSMCs are located in the arterial media, synthesizing the ECM and are responsible for the vessel contraction (72). A number of documents have provided evidence for the occurrence of senescence in VSMCs during atherosclerosis, such as lower level of proliferation capacity (93), higher expression levels of p16 and p21, and decreased telomere length (94) as well as larger and flattened morphology in comparison to cells separated from healthy arterial media (95). Senescent VSMCs in atherosclerotic plaques reduce the expression of proteins required for contraction, including α-smooth muscle actin (α-SMA), smooth muscle myosin heavy chain (SM-MHC) and calponin, but they increase production of inflammatory cytokines (96, 97). Furthermore, senescent human VSMCs in atherosclerotic lesions exhibit reduced expression of anti-inflammatory factors and secrete SASP mediators which induce chemotaxis of monocytes, expression of adhesion molecules and cytokines by ECs and promote atherosclerosis progression. Moreover, senescent human VSMCs can increase plaque vulnerability by producing lower amounts of collagen (98). In addition to senescent ECs and VSMCs, senescent immune cells, like macrophages and T cells, mainly contribute to the development of atheroma and progression of atherosclerotic plaques (99).

Senescent macrophages characterized by increased SA-β-GAL activity as well as p53 and p16 expression, display impaired cholesterol efflux and enhanced senescent-related atherosclerosis (100, 101). On the other hand Ox-LDL inhibits macrophages proliferation and migration, induces cellular senescence and promotes the secretion of inflammatory factors, such as TNF-α, monocyte chemoattractant protein-1(MCP-1), and IL-1β, which may create a positive feedback loop (102).

In addition to macrophages, senescent T cells with CD8^+^CD57^+^CD27^-^CD28^null^ phenotype, through producing large amounts of IFN-γ and TNF-α, promote inflammation and development of atherosclerosis (103). Furthermore, telomere shortening in T cells has been observed in patients with atherosclerosis. Terminal restriction fragments (TRF) analysis has shown that the mean length of TRF in leukocytes of CAD patients is shorter than control individuals who had no family history of CAD (104). It has been found that IFN-γ-producing CD28^null^CD4^+^ T cells accumulate in the heart-draining lymph nodes of aged mice and adoptive transfer of these cells results in proinflammatory responses in young mice (105, 106). It is also shown that senescent CD4^+^ T cells can infiltrate to heart and promote myocardial inflammation and stress response leading to age-related cardiac dysfunction (107). Additionally, the importance of senescent T cells has been reported in human hypertension. A higher frequency of CD57^+^CD28^−^CD8^+^ T cells as well as increased expression of CXCL11 has been reported in the patients with hypertension compared to healthy controls, suggesting a role for immunosenescent proinflammatory cytotoxic CD8^+^ T cells in hypertension (108). Interestingly, a higher frequency of senescent CD57^+^CD8^+^ T cells has been observed in patients with acute myocardial infarction (MI), which correlated with cardiovascular mortality 6 months after acute MI (109). However, it is still unclear whether accumulation of senescent T cells is the cause or the result of atherosclerosis. In this regard, it has been hypothesized that senescent T cells, through secreting large amounts of IFN-γ, are directly involved in the macrophages activation, metalloproteinases production, ECM destruction and thereby pathophysiology of atherosclerosis (110). In addition, senescent T cells may participate in VSMC and ECs lysis by producing large amounts of perforin and granzyme and lead to atherosclerosis progression (98).

## Drivers of immunosenescence and atherosclerosis

**Figure 1:**
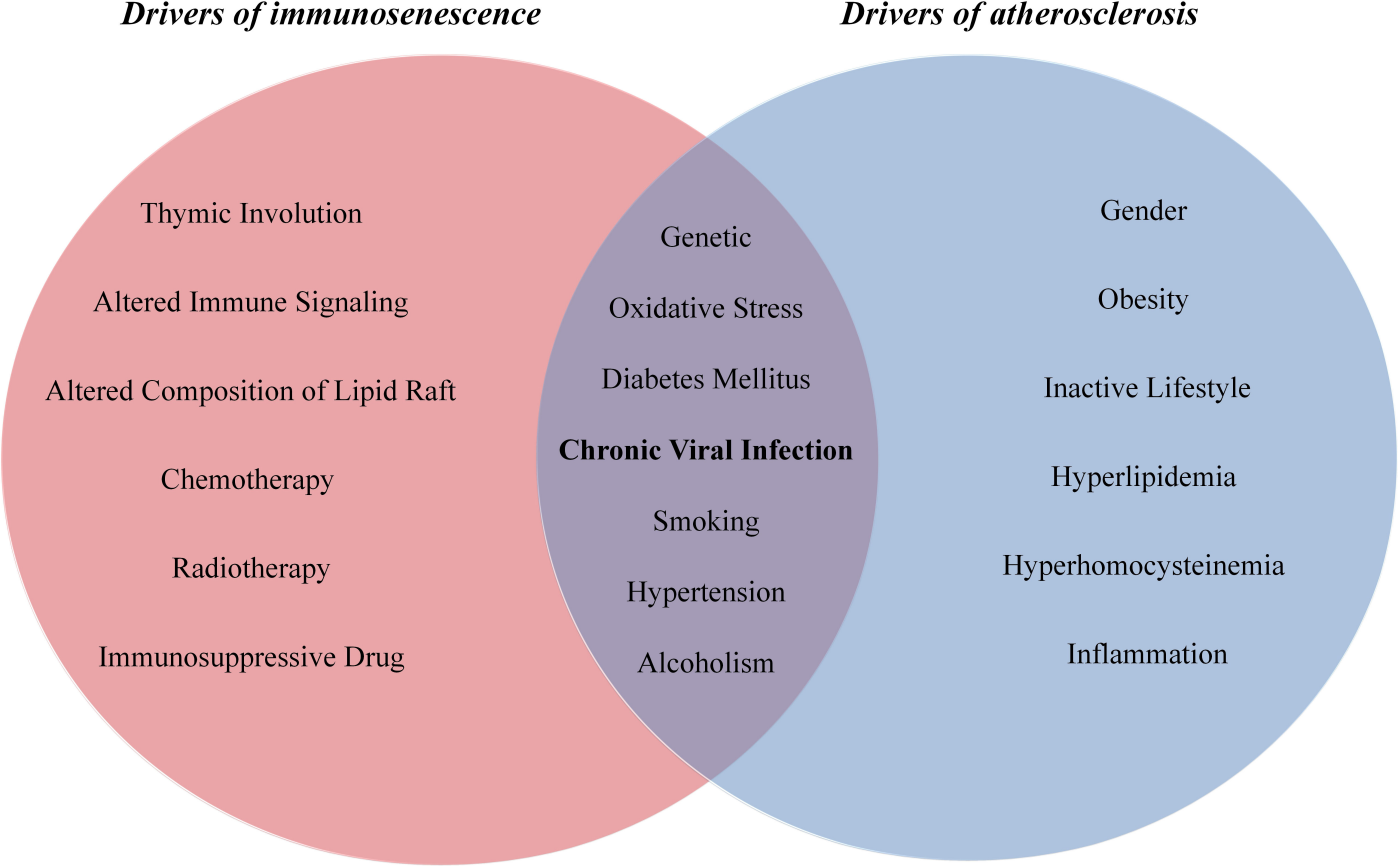
The drivers of immunosenescence and atherosclerosis.

Both immunosenescence and atherosclerosis are multifactorial conditions which share common stimulators, including Ox-LDL (111, 112), inflammation (113, 114), ROS (115, 116), cigarette smoke (117, 118), hypertension (119, 120), hyperglycaemia (121, 122), viral infection (123, 124), mitochondrial failure (125, 126) and genomic damage (127, 128) (**Figure 1**). Certainly, various drivers of senescence induce SASP secretion and thereby stimulate chronic and low-grade inflammation that participates in atherosclerosis development, and in turn, diverse stimulators of atherosclerosis induce cellular senescence and SASP production.

One of the most important causes of immunosenescence, which also may participate in atherosclerosis development/progression, is viral infection. Chronic viral infections can trigger cellular senescence directly and indirectly mainly *via* inducing DNA damage and prolonged cytokine signaling, respectively (129, 130). It has been shown that viruses, through induction of senescence affect proliferation capacity and function of T cells, quality, and quantity of innate and adaptive immune responses, recruitment of immune cells and secretion of proinflammatory mediators. Therefore, accumulation of viral-induced senescent cells in blood and tissues may contribute to senescence-associated inflammatory onditions, such as atherosclerosis (4, 131–134). Indeed, virus infections through different mechanisms can induce immunosenescence and atherosclerosis (**Table 1**). In the following sections, we will discuss different chronic human infections with DNA and RNA viruses which play parallel roles in the immunosenescence and atherosclerosis processes.

**Table 1:** Induction of atherosclerosis and immunosenescence by different viruses.

Virus	Atherosclerosis	Immunosenescence	References
**CMV**	Expression of cytokines and chemokines Expresion of cellular adhesion molecules Proliferation and migration of VSMCs Molecular mimicry Inhibition of apoptosis Inflammatory reactions Endothelial injury Coagulation and thrombosis Lipid accumulation	Decreased frequency of naïve T cells Decreased expression of CD27 and CD28 Increased expression of KLRG-1 and CD57 Re-expression of CD45RA Production of granzyme B Induction of inflammaging	**(** 135–138 **)**
**EBV**	Secretion of pro-inflammatory cytokines Expression of ICAM-1 Proliferation of VSMCs Induction of blood monocytes Alteration in lipid metabolism	Replicative stress G1 phase cellular arrest DNA damage Alteration in metabolic pathways	**(** 139–143 **)**
**HBV**	Alteration in macrophage phenotype? Induction of chronic inflammation Induction of fatty acid oxidation	Increased expression of p16 ^INK4a^ and p21 G1 phase cellular arrest DNA damage	**(** 144–148 **)**
**HCV**	Increased production of IL-1β and TNF-α Induction of chronic inflammation Increased synthesis of MMP-9 Generation of ROS	Decreased expression of CD27 and CD28 Increased expression of KLRG-1 and CD57 Increased expression of TIM-3 and P16^INK4a^ Telomere shortening	**(** 149–152 **)**
**HIV**	Production of inflammatory mediators Secretion of MCP-1/CCL2 chemokine Expression of adhesion molecules Activation the macrophage inflammasome Foam cell formation	Decreased CD4/CD8 ratio Increased CD28^−^CD8^+^ T cells Increased expression of TIM-3 and P16^INK4a^ Increased levels of IL-6 and TNFα Decreased T cell repertoire T cell Telomere shortening	**(** 153–156 **)**
**SARS-CoV-2**	Cytokine storm Endothelial injury Thromboinflammatory response Alteration in lipid metabolism	Increased expression levels of p16, p21, SA-β-Gal in ECs SASP production	**(** 157–160 **)**

### Cytomegalovirus

CMV is an enveloped, double stranded DNA β-herpesvirus which establishes lifelong latent infection in the population with sporadic reactivation in immunocompromised patients (161). The CMV infection/reactivation is mainly controlled but not eradicated by IFN-γ-producing CD4^+^ and CD8^+^ T cells in healthy immunocompetent individuals (162). Between 10-30% of memory T cells in the peripheral circulation of infected individuals are found specific to CMV epitopes (163, 164). CMV-specific CD8^+^ and CD4^+^ T lymphocytes display hallmarks of senescence defined by CD57 expression that is associated with decreased proliferation capacity and function of these cells (165). It is assumed that continued antigen exposure during CMV infection results in telomere erosion and replicative senescence generally in CD8^+^ cytotoxic T lymphocytes (CTL) (166–168). CMV-specific T lymphocytes also exhibit higher levels of proinflammatory cytokines, such as IFN-γ and TNF-α and lower proliferative responses upon stimulation, possibly due to the their telomeres shortening (169).

Recent cohort studies revealed that the excess mortality rate in CMV-seropositive elderly subjects is primarily related to senescence-associated vascular disease (135, 136). Several studies found relationships between increased inflammatory mediators and CVD-related deaths in CMV-infected elderly individuals (136, 170). It has been shown that CMV infection is correlated with increased risk of multiple senescence-associated comorbidities, especially diabetes, atherosclerosis, and CVDs (171). Furthermore, DNA and proteins of CMV have been found in human atherosclerotic plaques (137). It is also suggested that CMV can participate in atherosclerosis by inducing vascular endothelial dysfunction and apoptosis, reduced matrix metalloproteinase 9 (MMP9) activity, increased coagulation and thrombosis, elevated release of proatherosclerotic or proinflammatory molecules, increased intimal thickening and lipid deposition (138). Therefore, systemic inflammation as well as increased circulating senescent and proinflammatory CD8^+^ T cells during CMV infection may underlie proper condition for initiation and progression of atherosclerosis.

### Epstein-Barr virus

EBV is an enveloped double stranded DNA, B lymphotropic γ-herpesvirus that causes an acute infection known as infectious mononucleosis. However, EBV establishes a latent infection in human hosts that is associated with a variety of malignancies, including Extranodal NK/T-cell lymphoma, Burkitt lymphoma, Angioimmunoblast T-cell lymphoma, Hodgkin’s lymphoma and Post-transplant lymphoproliferative disease (172, 173). EBV specific humoral and cellular immune responses are particularly important for controlling and treating EBV-induced acute lymphoproliferative diseases (174). It has been found that EBV infection induces B cell hyper-proliferation *via* upregulating the viral latency proteins, EBNA2 and EBNA-LP, which results in replicative stress, DNA damage, activation of the DNA damage response (DDR) pathway and eventually, cellular senescence (139, 140, 175–177). A previous study reported that EBV-infected B cells trigger G1 phase cellular arrest (141). Even early EBV-infected B cells exhibit increased markers of OIS, including H3K9me3 senescence-associated heterochromatic foci as well as higher levels of p16, p21 and p53 (178). EBV-infected cells also undergo metabolic reprogramming such as decreased oxidative phosphorylation and purine nucleotide pools, which contribute to increased replication stress and establishment of persistent DNA damage (178). Additionally, antigen-specific T cells isolated from the peripheral blood of EBV and CMV positive patients have higher expression levels of several senescence-associated markers; including KLRG-1 while exhibit reduced antigen-specific T cell repertoire diversity (179). These data suggest that chronic EBV infection may participate in replicative and oncogene-induced cellular senescence.

The effect of EBV to increase the risk of atherosclerosis CAD has been studied in detail. Some studies showed higher levels of EBV-specific antibodies in patients with atherosclerosis (142, 180). Moreover, it has been found that following reactivation of latent virus, EBV-encoded deoxyuridine-5^’^-triphosphate nucleotidohydrolase (dUTPase) can induce proinflammatory cytokines secretion from peripheral blood monocytes which is associated with atherosclerosis progression (181). EBV may also be involved in the atherosclerosis by triggering IL-6 and TNF-α secretion by macrophages, intercellular adhesion molecule-1 (ICAM-1) expression on ECs and lipid profile alternations in blood (143).

### Hepatitis B virus

HBV is an enveloped, double stranded DNA virus of the Hepadnaviridae family that attacks the liver, triggering both acute disease and chronic disease such as cirrhosis, liver cancer and liver failure (182). Recently it has been found that 60% of hepatocellular carcinomas (HCCs) and more than 80% of liver cirrhosis cases exhibited features of replicative senescence as compared to 10% in normal liver (183, 184). In addition, there is higher level of SA-β-Gal activity in cirrhotic hepatocytes as compared with large-cell dysplasia (144, 184). Elevated expression levels of p21, reduced telomere length and lower levels of S, G2 and M phase markers in hepatocytes of chronic HBV-infected patients is also reported which correlated with liver fibrosis (185). Furthermore, in chronic human infection, HBV inhibits Top 1 protein and leads to topological DNA damage and telomere attrition in CD4^+^ T cells (145). Therefore, Top 1 inhibition by the virus is correlated with premature T cell immunosenescence (145). Several studies indicated that HBx protein of HBV, a transcriptional transactivator for virus replication, can carry out pro-senescent roles by increasing the expression levels of p16 (INK4a) and p21 (Waf1/Cip1) and reducing phosphorylation of Rb (146, 186). Additionally, increased secretion of SASP components, including IL-6 and angiogenin-2, has been reported in chronic HBV infection (187).

The association between HBV infection and CVDs remains controversial. Recent reports revealed that patients with HBV infection exhibited more subclinical atherosclerosis and carotid plaques as compared with non-infected controls (188, 189). A significant association between liver damage, as an independent factor, and development of subclinical atherosclerosis has also been shown (190). A previous study showed that HBV surface antigen (HBsAg) is a major contributor of atherosclerosis (147), but two other studies found no significant association between chronic HBV infection and development of carotid atherosclerotic plaques (191, 192). Moreover, a significant negative correlation between serum levels of triglycerides and HBV infection is also reported (148). In contrast, results of a meta-analysis study represented that exposure to HBV led to increase atherosclerosis-associated morbidity rate (193).

### Hepatitis C virus

HCV is an enveloped, positive sense single-stranded RNA virus that causes liver inflammation, leading to serious liver damage and hepatitis (194). Chronic HCV is a long-lasting infection, ranging in severity from a mild illness to a serious disease including liver cirrhosis and cancer (195). Several studies reported that chronic HCV infection can trigger cellular senescence by inducing higher concentrations of ROS, secretion of proinflammatory cytokines and growth factors, G1 cell cycle arrest, DNA damage, as well as epigenetic modifications of DNA and histones (149, 150, 196–200). In addition, it has been found that HCV-associated liver inflammation can promote the telomere shortening process and finally replicative senescence and HCC (184). Isolated memory T cells from HCV-positive patients exhibit shorter telomeres compared to healthy subjects (201). Additionally, CD8^+^ T cells isolated from peripheral blood of HCV-infected subjects show higher levels of DNA damage and hypophosphorylated signal transducer and activator of transcription 1 (STAT1) and STAT5 in response to IL-6 or IL-2 stimulation, respectively (202). Altogether, these data suggest that chronic HCV infection results in cellular senescence, and since these senescent cells are non-functional, they may predispose HCV-infected individuals to HCC (203).

Growing evidence suggests metabolic reprogramming and chronic hepatic and systemic inflammation induced by HCV can be involved in the development of atherosclerosis (204–207). It has been shown that HCV can live and replicate in the carotid plaques and is associated with arterial inflammation through increased levels of proatherogenic chemokines and cytokines as well as triggering proatherogenic metabolic factors (151, 208, 209). Furthermore, structural and non-structural proteins of HCV induce ROS generation and interfere with glucose and lipid metabolism leading to chronic inflammation, insulin resistance (IR), diabetes and fatty liver, which are known as major atherosclerosis risk factors (152). A higher TNF-α/adiponectin ratio in HCV-infected subjects is shown to be correlated with the development of IR and atherosclerosis (210). A recent multicenter study reported that direct-acting antiviral agents (DAAs) therapies in HCV-infected patients led to eradication of HCV as well as improvement in carotid atherosclerosis, reduction in carotid thickness and alternation in patients’ plaques value (211). The most surprising results of cohort studies have been published recently in which DAA treatments significantly reduced the risk of CVD outcomes in HCV-infected patients (212, 213). Therefore, a strong association between HCV infection and the atherosclerosis process exists.

### Human immunodeficiency virus

HIV is an enveloped, diploid positive sense single-stranded RNA retrovirus from Retroviridae family that mainly attacks CD4^+^ T cells and results in progressive loss of T cell subsets. HIV infection results in immunodeficiency, increased susceptibility to opportunistic infections as well as certain types of cancer such as Kaposi sarcoma and B cell lymphoma, and ultimately a syndrome defined as acquired immune deficiency syndrome (AIDS) (214). Recently, it has been found that HIV-infected individuals display T cell properties similar to those identified in elderly; i.e. decreased expression of CD28, lower naive/memory T cell ratios and hyporesponsiveness to vaccine (215). Growing evidence revealed higher expression level of p16 in active HIV-infected subjects as compared with healthy controls that is not correlated with the age of patients, suggesting that HIV infection is associated with cellular senescence. Also, anti-retroviral therapy (ART) decreased the expression levels of p16 in the CD4^+^ T cells population compared to healthy controls (153, 154). Moreover, results of a cohort study elucidated that corneal ECs of HIV-infected patients exhibit features of senescence, including decreased cell density as well as variation in cell size and shape as compared with the uninfected subjects (216). In a cohort in sub-Saharan Africa, higher expression levels of p16 and decreased telomere length in peripheral blood leukocytes of HIV infected patients is found (217).

In view of persistent inflammation and immune activation, HIV-positive individuals may have increased risk of HIV-related comorbidities and atherosclerosis-related risk factors such as hypertension, diabetes, and dyslipidemia (218, 219). The higher levels of IL-6 protein and mRNA in HIV-infected individuals can stimulate the production of several acute phase proteins, including C-reactive protein (CRP), serum amyloid A (SSA) and fibrinogen. The elevated levels of these inflammatory mediators have been associated with increased cardiovascular mortality in patients with HIV (155, 220, 221). Moreover, it has been reported that the HIV Tat protein induces endothelial dysfunction and MCP-1 secretion in porcine coronary arteries (156, 222). The gp120 protein of HIV has also been found to increase the levels of TNF-α and subsequently retention and oxidation of LDL in the arterial intima (223). The HIV Nef protein is also identified as an activator of macrophages by increasing CD36 expression, and it promotes macrophages transformation to foam cells through decreasing cholesterol efflux from these cells (224, 225).

### Severe acute respiratory syndrome coronavirus 2

SARS-CoV-2 is an enveloped, positive-sense, single-stranded RNA, highly infectious beta-coronavirus, which has been described as the causative agent of upper respiratory tract infections and subsequently coronavirus disease 2019 (COVID-19) (157). A recent study found that COVID-19 patients exhibited features of senescence in their airway mucosa (157). Increased secretion levels of SASP factors in patients with COVID-19 resulted in complement mediated lysis, SASP-induced paracrine senescence of ECs, macrophage and neutrophil infiltration, endothelial damage and extensive thrombosis in SARS-CoV-2- infected lung tissues (157). It has been found that SARS-CoV-2 leads to increased expression levels of senescence markers such as p16, p21 and Lamin B1(LMNB1) as well as SASP factors in cultured human bronchial epithelial cells 14 days after infection when the virus was undetectable (158). These results suggest that SARS-CoV-2 can provoke paracrine senescence through sustained production of virus-induced inflammatory mediators, even after SARS-CoV-2 is no longer detectable (158). Furthermore, other studies indicated that administration of senolytic drugs, including Navitoclax, Dasatinib and Quercetin can inhibit cellular senescence and therefore alleviated COVID-19-related lung disease and reduce inflammation in SARS-CoV-2-infected hamsters and mice (157, 158, 226). Additionally, it has been indicated that the spike protein of SARS-CoV-2 stimulates senescence phenotype in ECs by increasing p16, p21, SA-β-Gal (158).

COVID-19 infection is primarily considered as a destructive disease of the respiratory system, but its complications also lead to the cardiovascular system damage and occurrence of a variety of CVDs, including myocarditis, myocardial damage, heart failure and myocardial infarction (227, 228). It has been found that angiotensin-converting enzyme 2 (ACE2), one of the most important receptors for SARS-CoV-2 entry, is expressed on the cardiomyocytes (229). Furthermore, higher serum levels of troponin and N-terminal pro-brain-natriuretic peptide (NT-proBNP) were reported in severe COVID-19 patients, which were associated with more disease manifestation and mortality rate (159). Another observational study revealed the effect of SARS-CoV-2 infection on lipid metabolism and atherogenesis (22). In this regard, higher serum levels of free fatty acids, lysophosphatidylcholine, lysophosphatidylethanolamine, phosphatidylglycerol and subsequently increased presence of CVDs in recovered SARS infected patients were found (230). Moreover, two other studies represented that SARS-CoV-2 infection, through activating the coagulation pathway, secreting inflammatory cytokines and chemokines by ECs, inducing production of fibrinogen, antithrombin and D-dimers and eventually triggering disseminated intravascular coagulopathy (DIC), may affect the onset or development of atherosclerosis in severe COVID-19 individuals (231, 232). The induction of senescence in immune cells, especially in patients with Long COVID, however, remains to be investigated.

### The role of viral co-infections in the immunosenescence and atherosclerosis

The interplay between different viral co-infections and their synergistic effect on immunosenescence has been under investigation, recently. Viral co-infections such as in the case of CMV or HCV infection with HIV synergistically increase immunosenescence progression in patients (233–235). A previous study showed decreased telomere length within CD4^+^, CD8^+^ and immature (CD27^+^CD57^+^) T cell subsets in HIV/HCV co-infected Injecting Drug Users (IDU) as compared to HCV mono-infected and healthy subjects (233). This report is in line with the higher level of IL-6 in the same group which independently correlated with HIV/HCV co-infection and increasing age (236). Moreover, higher expression levels of immunosenescence markers, CD57 and P16^INK4a^, were observed in the liver tissue of HIV/HCV co-infected individuals compared to healthy controls (237). In addition, HIV co-infection increases DNA methylation and accelerated epigenetic ageing in chronic HCV patients compared to mono-infected patients (238). Similarly, higher levels of inflammatory markers such as IP-10, TNF-RII, and D-dimer are elevated in HIV/CMV co-infected individuals compared to HIV-mono-infected subjects or healthy controls (235).

In a similar manner, mounting evidence suggest that chronic viral co-infections may accelerate atherosclerosis. A cross sectional study showed higher prevalence of subclinical carotid plaque in relatively young (aged 46 years) HIV/HCV co-infected patientscompared to HIV mono-infected patients (239). Moreover, higher soluble vascular CAM-1 (sVCAM-1) and intercellular CAM-1 (sICAM-1) levels, as pro-atherosclerotic inflammatory biomarkers, have been reported in HIV/HCV co-infected patients as compared to HIV mono-infected patients (240). Increased morbidity and mortality rate and subclinical carotid artery diseasein HIV/HCV co-infected women were also associated with higher CMV IgG levels (241). However, another study on women found that HIV/HCV co-infection was not associated with greater carotid artery intima-media thickness (242).

### Chronic viral infections, immunosenescence, CVDs and premature biological ageing

The premature biological ageing process is defined as age-related changes in function and composition of the human body. This process is characterized by chronic systemic immune activation, elevated inflammatory and coagulation markers (243, 244), decreased leukocyte telomere length (217), mitochondrial DNA mutations (245), cell senescence, impaired autophagy (246), epigenetic alterations and higher DNA methylation levels (247), which are correlated with onset of age-related comorbidities such as diabetes, CVDs, or obesity (248). Chronic viral infections are probably one of the most important drivers of premature biological ageing. Previous studies found increased age acceleration and higher DNA methylation in both blood cells and brain tissues of chronic HIV-infected patients compared to HIV-negative individuals (247, 249, 250). Another study on PBMCs of 122 nonagenarians and 21 young controls represented increased DNA methylation in chronically CMV infected individuals irrespective of age. Their findings also indicated a significant correlation between CMV seropositivity and higher epigenetic age in both groups (251). Another study confirmed a positive correlation between the host cell methylation state and CMV seropositivity, as well (252).

Such ageing related epigenetic alterations were also reported in COVID-19 survivors under 60 years of age (253). These individuals represented accelerated telomere shortening and increased DNA methylation in CpG islands of leukocytes and lymphocytes (253). This is noteworthy in the sense that SARS-CoV-2 infection is currently considered an acute viral infection.

The accelerated biological ageing induced by viruses can be a risk factor for vascular diseases and the subsequent cardiovascular and cerebrovascular events (254). The associations between age-related telomere shortening and CAD as well as CVD-related mortality have already been investigated (255–257). In addition, structural alterations in biologically ageing arteries such as elastin fragmentation (258), collagen accumulation (259), decreased vascular compliance (260) and increased arterial stiffness (261) as well as elevated blood pressure (262) are reported as the consequence of chronic viral infections, too. Premature ageing may impose similar risks of developing CVDs in younger individuals. In this regard, a previous study investigated the association between biological age acceleration and carotid intima-media thickness, in a 21-year follow-up cohort. Their results showed that 5-years increase in the epigenetic age in the whole blood was related to approximately 0.01 mm greater carotid intima–media thickness suggesting a potential contribution of biological age acceleration to the development of CVD (263). A shorter telomere length of the buccal epithelium in CVD patients is also shown to be associated with biological ageing (264).

## Conclusion

**Figure 2:**
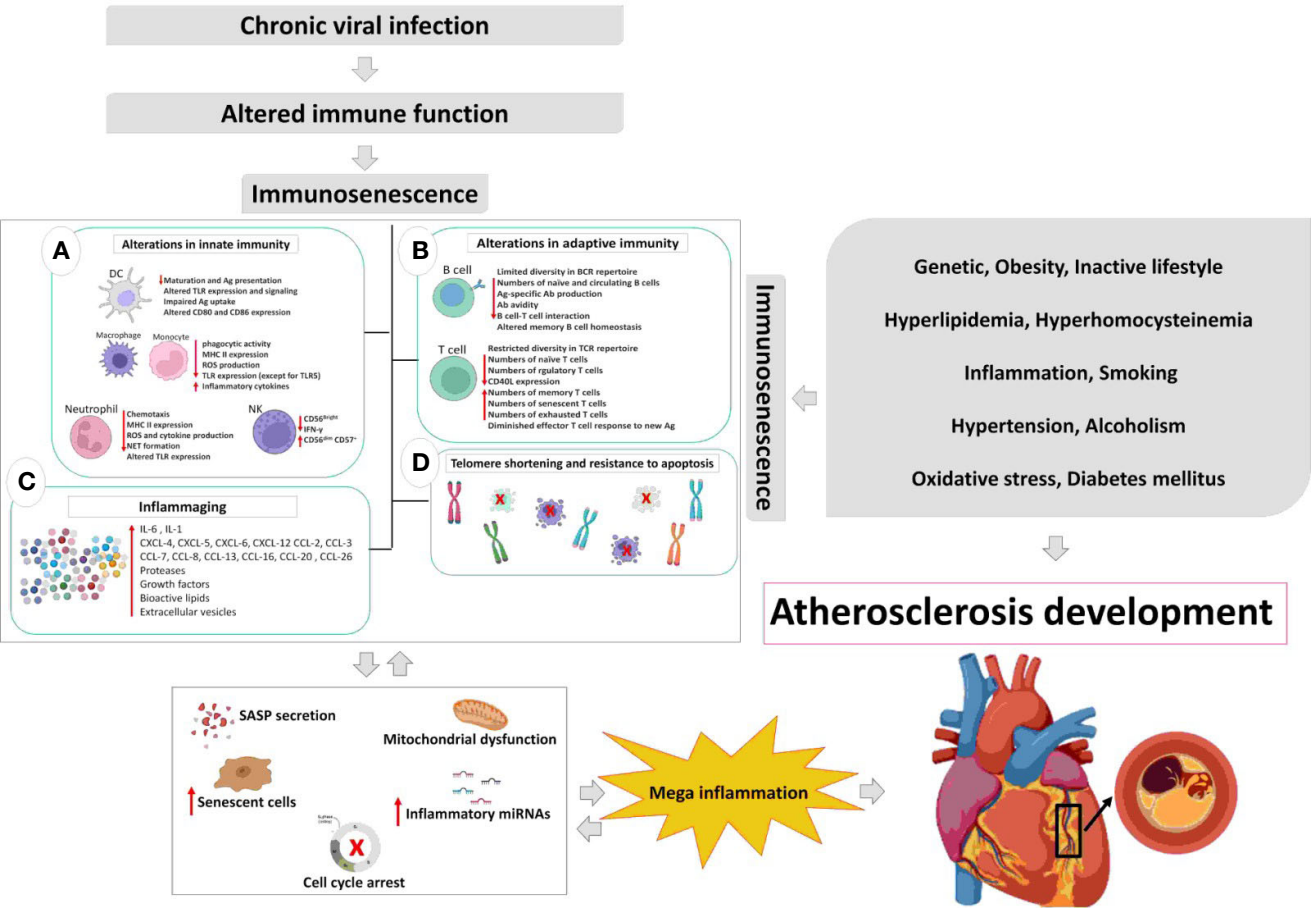
The relation between chronic viral infection-induced immunosenescence and atherosclerosis development. Chronic viral infections modify immune responses and immune functions over the time course of infection. The hallmark of most chronic viral infections is inflammaging, which triggers cell cycle arrest, SASP secretion, mitochondrial dysfunction, telomere shortening, and immunosenescence. Such alterations are accompanied by acceleration of atherosclerosis even in younger adults, thereby; in a positive feedback loop inflammaging accelerates atherosclerosis and ageing of the immune system. DC, dendritic cell; Ag, antigen; TLR, toll-like receptor; MHC, major histocompatibility complex; ROS, reactive oxygen species; NET, neutrophil extracellular traps; NK, natural killer; BCR, B cell receptor; Ab, antibody; TCR, T cell receptor; SASP, senescence-associated secretory phenotype.

Immunosenescence is the hallmark of many inflammatory and autoimmune diseases that is stimulated by multiple factors. Recent data indicate that chronic viral infections manipulate the pathways involved in replicative senescence (RS), oncogene-induced senescence (OIS), and possibly genotoxicity-induced senescence (GIS) in immune and non-immune cells. The senescence pathways induced by infectious agents are shared with other senescence inducing stimuli. The induction of senescence in immune cells is more robust in chronic viral infections due to direct stimulation of the immune system by viral antigens. From early childhood, the immune cells of human-beings are challenged with viral infections and fortunately enough, in most cases the virus is contained and even eradicated by immune system. However, continuous encounter with viruses and especially establishment of chronic viral infections in the body results in a state of more inflammatory and less protective immune response. In atherosclerosis, as one of the old inflammatory conditions and the mother of cardiovascular and cerebral stroke, immunosenescence is induced both in immune and non-immune cells. Therefore, chronic viral infections, through induction of immunosenescence, may directly or indirectly play a role in development or progression of atherosclerosis (**Figure 2**). The premature ageing as a result of viral co-infections may also accelerate immunosenescence and inflammatory diseases. In this article, we reviewed evidence of the possible role of chronic viral infections and co-infections in the induction of immunosenescence as a contributor in atherosclerosis.

## Author contributions

AGT wrote the first draft of the manuscript. MD conceptualized, designed the study and critically revised the manuscript. All authors reviewed and approved the final version of the manuscript.

## Funding

This study was supported by grants no. 93-7072, and 97-17106 from Shiraz University of Medical Sciences, Shiraz Iran.

## Acknowledgments

The authors would like to thank all the individuals who participated in the mentioned studies by donating blood, tissue, etc. without whom these knowledge would have not been available.

## Conflict of interest

The authors declare that the research was conducted in the absence of any commercial or financial relationships that could be construed as a potential conflict of interest.

## Publisher’s note

All claims expressed in this article are solely those of the authors and do not necessarily represent those of their affiliated organizations, or those of the publisher, the editors and the reviewers. Any product that may be evaluated in this article, or claim that may be made by its manufacturer, is not guaranteed or endorsed by the publisher.
